# Multiple vaccinations with UV- attenuated cercariae in pig enhance protective immunity against *Schistosoma japonicum *infection as compared to single vaccination

**DOI:** 10.1186/1756-3305-4-103

**Published:** 2011-06-10

**Authors:** Dandan Lin, Fang Tian, Haiwei Wu, Yanan Gao, Jingjiao Wu, Donghui Zhang, Minjun Ji, Donald P McManus, Patrick Driguez, Guanling Wu

**Affiliations:** 1 Department of Pathogen Biology & Immunology, Nanjing Medical University, Nanjing, Jiangsu, 210029, China; 2 Jiangsu Province Key Laboratory of Modern Pathogen Biology, Nanjing, Jiangsu, 210029, China; 3 Jiangxi Provincial Institute of Parasitic Diseases, Nanchang, Jiangxi, 330046, China; 4 Department of Pathogen Biology & Immunology, Yangzhou University Medical College, Yangzhou, Jiangsu, 225001, China; 5 Molecular Parasitology Laboratory, Queensland Institute of Medical Research, 300 Herston Road, Brisbane, QLD 4029, Australia

## Abstract

**Background:**

Schistosomiasis japonica is a major public health problem in the endemic areas of China, the Philippines, and Indonesia. To date, a vaccine has not been developed against this disease but immunization with UV-attenuated cercariae can induce a high level of protective immunity in Landrace/Yorkshire/Duroc crossbred pigs. To compare the efficacy of a single vaccination and multiple vaccinations with UV-attenuated *Schistosoma japonicum *cercariae, two groups of pigs received either one or three exposures to 10,000 cercariae attenuated with 400 μw UV.

**Results:**

Pigs with a single immunization had a 59.33% reduction in adult worm burden, a 89.87% reduction in hepatic eggs and a 86.27% reduction in fecal eggs at eight weeks post-challenge (*P *< 0.01). After three immunizations, protection increased to 77.62%, 88.8% and 99.78% reduction in adult worms, hepatic eggs and fecal eggs, respectively (*P *< 0.01). Humoral and cellular immunological parameters measured indicated that schistosome-specific IgG1 and IgG2 levels in the vaccinated groups were higher than in the infection-control group. Triple vaccinations resulted in higher levels of antibodies, especially IgG2, compared with a single vaccination and IFN-γ levels increased with repeated immunization with UV-irradiated cercariae.

**Conclusion:**

The high levels of protection against *S. japonicum *infection can be achieved with a UV-attenuated vaccine in pigs, and that three vaccinations were possibly more effective than a single vaccination. Moreover, triple vaccinations evoked a more vigorous IFN-γ response and a stronger antibody-mediated response, especially an increase in the levels of IgG2 antibodies.

## Background

Despite decades of intense efforts to control schistosomiasis japonica, the disease is still a major public health problem in China, the Philippines, and Indonesia. Schistosomiasis japonica is a zoonosis that can be spread through a variety of wild or domestic reservoir hosts including bovines and swine [1]. Although comprehensive measures, including community chemotherapy, snail control and environmental modifications are important for reducing the prevalence and morbidity in areas of endemicity, reinfection is very difficult to control [2]. Therefore, development of vaccines to protect both human and the domestic animals is an attractive goal.

It is well recognized that the radiation-attenuated (RA) vaccine can induce high and stable protection against *Schistosoma mansoni *challenge in many animal models, including mice and primates [3]. Both antibody and CD4^+ ^T-cell-mediated, IFN-γ-dependent effector mechanisms have been demonstrated in the mouse model against *S. mansoni *[3]. In contrast, with *S. japonicum*, the protection levels induced by RA vaccines in mice reported by many laboratories were markedly different. Moloney *et al *[4] considered that mice could be partially protected against *S. japonicum *by prior exposure to UV-attenuated infections. However, Zhang *et al *[5] and Osada *et al *[6] reported that gamma-irradiatied cercariae provided a lower level of protection (3.7~24%) in C57BL/6 mice, and our previous experiments also showed that the RA vaccine could only produce protection levels of 2.27~38.67% in C57BL/6 mice and failed to effectively induce a Th1 response [7,8]. Thus, studies from different laboratories have shown that protection in mice induced by attenuated *S. japonicum *cercariae is variable. In contrast, the pig is not only a significant reservoir host of *S. japonicum*, but being a large animal with close biological similarities to humans, it provides a better experimental model than the mouse to study the relevant immune events associated with protection [9-13]. In artiodactyls, RA vaccination induces consistently high levels of protection against *S. japonicum *infection, being above 60% in pigs and cattle [14-18]. Therefore, experimental studies on porcine schistosomiasis japonica can provide novel information about how to make an effective and feasible vaccine applicable to the field.

Our previous studies on pigs indicated that a single immunization with radiation-attenuated *S. japonicum *cercariae was able to induce 63.8% and 71.8% reductions in worm burden and hepatic eggs, respectively [19]. In this study, we undertook further vaccination experiments to evaluate the protective efficacy in pigs following single and triple vaccination with UV- attenuated *S. japonicum *cercariae, and compared the humoral and cellular immune responses generated.

## Results

### 1. A high level of protection against *Schistosoma japonicum *induced by UVAC vaccination

#### 1.1 Lower adult worm and liver egg burdens in vaccinated pigs

**Figure 1:**
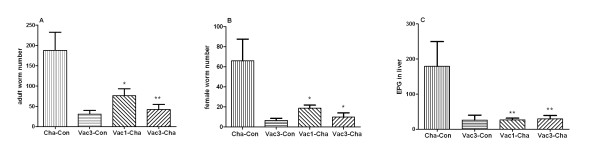
**The number of adult worms, female worms and hepatic eggs per gram in all groups**. Figures 1A, 1B and 1C show the average number of adult worms, female worms and liver EPG for each group, respectively. Cha-Con group was only challenged with *S. japonicum *normal cercariae. Vac3-Con group was vaccinated with UVAC three times at 4-week interval. Vac1-Cha group was vaccinated with UVAC at week 8 and challenged with normal cercariae at week 12. Vac3-Cha group was vaccinated with UVAC three times at 4-week interval and challenged with normal cercariae at week 12. * *P *value < 0.05 and ** *P *value < 0.01 compared to Cha-Con group. Values depicted are means ±SEM for results from six animals.

The number of adult worms recovered and hepatic eggs per gram at 8 weeks post-challenge are shown in Figure 1. The Vac3-Con group had a mean of 31 worms, suggesting that the vaccinating parasites were not completely attenuated and some could develop to adult worms and even produce eggs. The accumulative number of surviving worms from triple vaccinations is around 3% of the number of challenging worms. The escaping cercariae from a single vaccination are likely to be lower than this figure. Therefore, the numbers of escaping cercariae from vaccinations were not more enough to significantly affect the evaluation of vaccination efficacy. To coordinately compare the protection efficiencies of single or triple vaccinations with UVAC, we ignored the "escaping worms". The single vaccination group (Vac1-Cha) had a 59.33%, 71.71% and 89.87% reduction in worm number, female worm number and hepatic egg burden, compared to the challenge-control (Cha-Con) group. Similarly, the triple UVAC vaccination protocol (Vac3-Cha) resulted in 77.62%, 85.35% and 88.8% reductions in worm number, female worm number and liver eggs, respectively. Because the numbers of worms in Vac3-Cha and Vac1-Cha groups include surviving worms from vaccinations, the calculated protection efficacies likely reflect slight underestimation of true protection levels. Furthermore, there was no statistically significant difference between the Vac1-Cha and Vac3-Cha groups in the number of total worms, female worms and EPG in the liver. Thus, vaccination once or three times with UVAC both induced high level protection against *S. japonicum *challenge.

#### 1.2 Lower egg output in feces of the vaccinated pigs

**Figure 2:**
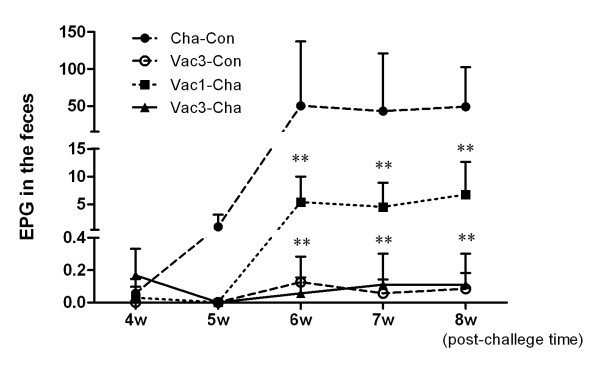
**Fecal egg output in the vaccinated and control pig groups after *Schistosoma japonicum *cercariae challenge**. **P value < 0.01 for the Vac3-Cha group or Vac1-Cha group compared with the Cha-Con group. Values depicted are means ±SEM for results from six animals.

Fecal egg output in the UVAC vaccinated groups was significantly less than the infection control group from 6 weeks post-infection onwards. At eight weeks post-challenge there was a 99.78% reduction in the Vac3-Cha group and a 86.27% reduction in the Vac1-Cha group, compared with the Cha-Con group. The lowest number of fecal eggs was found in the Vac3-Con groups (Figure 2).

### 2. Humoral response against *Schistosoma japonicum *infection

#### 2.1 High levels of IgG antibodies induced after triple vaccinations with UVAC

**Figure 3:**
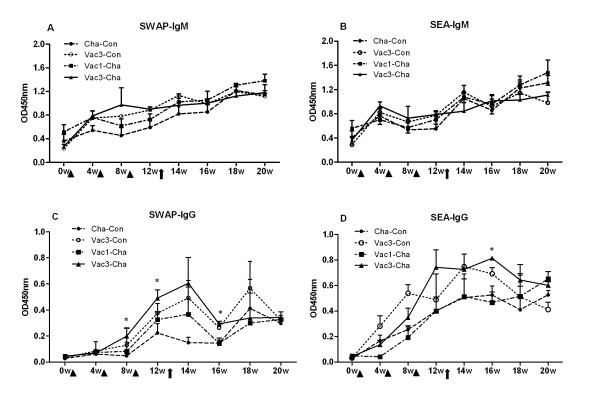
**Schistosome antigen-specific IgM and IgG responses in different swine groups**. Vac3-Cha and Vac3-Con groups were immunized with UVAC at weeks 0, 4 and 8. The Vac1-Cha group was immunized only at week 8 (triangles). The Vac3-Cha, Vac1-Cha and Cha-Con groups were challenged with normal cercariae at week 12 (arrow). *P < 0.05 for the triple vaccinated compared with the Cha-Con group. Values depicted are means ±SEM for results from six animals.

As shown in Figure 3, immunization with UVAC followed by challenge with normal cercariae induced schistosome antigen-specific IgM and IgG responses in pigs. And schistosome-specific IgM levels in the Vac3-Cha group developed more rapidly than IgG antibodies. While SWAP- and SEA-reactive IgM antibody titers in the vaccinated groups were similar with those in the control groups, triple UVAC vaccinations induced pigs to produce a stronger IgG antibody response than a single UVAC immunization, particularly during the pre-challenge and early post-challenge stages of the time course.

#### 2.2 High levels of IgG1 and IgG2 isotypes, especially IgG2 isotype, induced by triple UVAC immunizations

**Figure 4:**
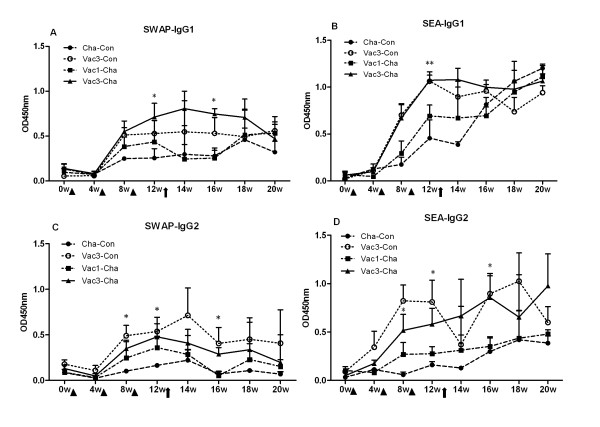
**Schistosome antigen-specific IgG1 and IgG2 responses in the different swine groups**. All groups were as described in Figure 4. *P < 0.05 and **P < 0.01 for the Vac3-Cha group compared with the Cha-Con group. Values depicted are means ±SEM for results from six animals.

The levels of SWAP-, SEA-specific IgG1 and IgG2 gradually increased after immunization (Figure 4). Before *S. japonicum *challenge, the levels of schistosome-specific IgG1 and IgG2 in the vaccinated pigs in Vac3-Cha, Vac3-Con, and Vac1-Cha groups were much higher than those in naïve pigs in the Cha-Con group. Moreover, triple UVAC vaccination (Vac3-Cha and Vac3-Con) led to stronger induction of schistosome specific IgG1 and IgG2 than a single immunization. Within the Cha-Con group, SEA-IgG1 increased rapidly post-challenge as eggs start lodging in the tissues and even exceeded the levels in the other three groups by the end of the experiment. In contrast, SEA-IgG2 in Cha-Con group maintained the lowest levels throughout the time course. The IgG1/IgG2 ratios of the Vac3-Cha and Vac1-Cha groups post-challenge were usually lower than those in the Cha-Con group with the lowest levels observed in the Vac3-Cha group. Thus, the higher levels of IgG2 antibody were likely the result of the UVAC vaccination and the number of immunizations given (Table 1).

**Table 1:** Comparison of IgG1/IgG2 ratio among the four trial groups

Week	SWAP-specific IgG1/IgG2				SEA-specific IgG1/IgG2			
	
	Cha-Con	Vac3-Con	Vac1-Cha	Vac3-Cha	Cha-Con	Vac3-Con	Vac1-Cha	Vac3-Cha
0w	-	0.294	-	1.050	-	0.235	-	1.254
4w	-	0.538	-	1.648	-	0.322	-	0.562
8w	-	1.044	1.554	1.577	-	0.855	1.091	1.300
12w	1.541	0.983	1.207	1.490	2.864	1.312	2.524	1.847
14w	1.342	0.766	0.852	1.968	3.023	2.429	2.152	1.619
16w	4.014	1.310	4.902	2.591	2.732	1.070	1.983	1.165
18w	4.232	1.092	2.246	2.099	2.531	0.720	2.176	1.497
20w	4.642	1.376	3.496	2.382	3.118	1.572	2.323	1.089

### 3. A predominant IFN-γ response in the Vac1-Cha group and a mixed IFN-γ/IL-10 response in the Vac3-Cha group during the early stages of the infection

**Figure 5:**
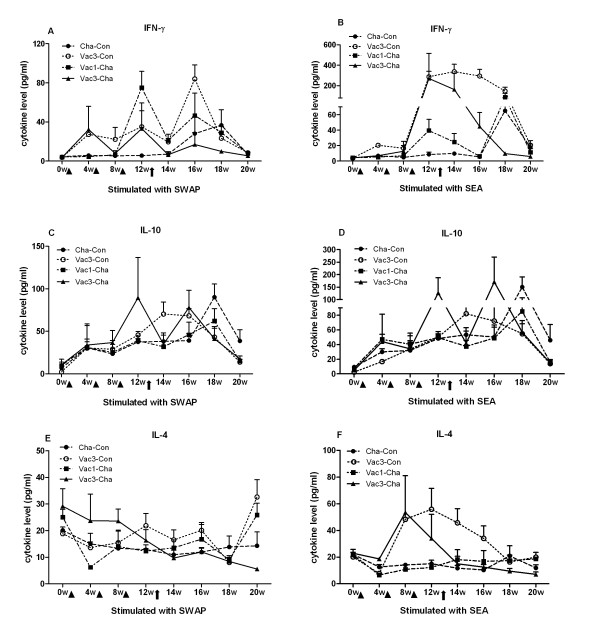
**IFN-γ, IL-10 and IL-4 produced by PBMC stimulated with SWAP and SEA in the 4 groups of pigs**. Triangles and the arrow on the X-axis show the time of immunization(s) and infection, respectively. Values depicted are means ±SEM for results from six animals.

The production of the three cytokines, IFN-γ, IL-10 and IL-4, in the PBMC supernatants was investigated throughout the trial (Figure 5). In the Vac3-Cha group IFN-γ production by PBMC, stimulated with either SWAP or SEA, increased after the UVAC immunizations with the highest levels in pre-challenge SEA-stimulated PBMC. As the challenge infection developed and egg deposition in the tissues increased, IFN-γ production was rapidly down-regulated with the lowest levels at 6 weeks post-infection. Similarly, single UVAC immunization induced the PBMC to produce IFN-γ after SWAP or SEA stimulation with peaks prior to challenge infection. Thus, vaccination with UVAC once or three times could both induce the production of IFN-γ prior to *S. japonicum *challenge infection. However in contrast to the triple UVAC vaccination group, at week 6 post-challenge, SEA-pulsed PBMC from the Vac1-Cha group produced high levels of IFN-γ. IL-10 secretion by PBMC after SWAP or SEA stimulation in the Vac3-Cha group increased with the subsequent vaccinations peaking at 12 weeks. The IL-10 response to SWAP and SEA stimulation was not different between the Vac1-Cha and Cha-Con groups before *S. japonicum *challenge but with the start of egg laying both increased, peaking at 6 weeks post-challenge. At this time the IL-10 level in the Cha-Con group was highest among the four groups. Finally, SWAP-stimulated IL-4 levels from the Vac3-Cha, Vac1-Cha and Cha-Con groups were low and showed no significant difference before the challenge infection. At the pre-challenge time point, SEA-pulsed PBMC from pigs in the triple vaccinated group produced the highest amount of IL-4 compared to the other two groups but this decreased in Vac3-Cha after challenge. As a control, PBMC from all groups in trial stimulated with PHA produced the highest levels of all three cytokines while in the absence of any antigenic stimulation negligible levels were detected (data not shown).

## Discussion

This study has demonstrated that Landrace/Yorkshire/Duroc crossbred pigs immunized with 400 μw UV-radiated *S. japonicum *cercariae develop significant resistance against normal cercarial challenge. UVAC vaccination elicited a significant reduction in worm burdens and in egg numbers in the livers and feces of vaccinated pigs relative to the challenge control group. Moreover, although there is no statistically significant difference between the Vac1-Cha and Vac3-Cha groups in the number of total worms and EPG in the liver, our data strongly suggested that triple vaccinations with the UVAC vaccine could lead to more destruction of adult worms and/or reduced development of juveniles, and lower egg release to the environment 77.62% and 99.78% reduction in worm burdens and EPG in feces, respectively than a single immunization 59.33% and 86.27% reduction in worm burdens and EPG in feces, respectively. These results are consistent with previously published results with attenuated *S. japonicum *cercarial vaccines. Protection against *S. japonicum *in pigs has been reported to reach 56%~95% worm reduction after ultraviolet-attenuated cercariae vaccination and 95% worm reduction in pigs vaccinated with γ-attenuated cercariae [15,17,18]. Similarly, in cattle, after vaccination with ultraviolet-attenuated cercariae, there was a 89% reduction in worm burden and 65.1%~75.6% worm reduction with the γ-irradiated cercarial vaccine [16,20]. Previous experiments have also shown that higher protection against *S. japonicum *infection is achieved after triple exposures to irradiated cercariae. In pigs, protection in regards to reduced worm burdens increased from 86% to 95% after three UVAC immunizations compared to a single immunization [15]. The levels of protection (reduction in adult worm numbers) were 61.4%, 74.6% and 84.1% in cattle after immunization with the X-ray irradiated cercarial vaccine delivered for one, two and three times, respectively [21]. For *S. mansoni*, multiple vaccinations with radiation-attenuated cercariae have also been shown to induce a higher level of protection than a single vaccination. In the grivet monkey, *Cercopithecus aethiops*, protective efficacy was highest (48%) after three vaccinations, and in baboons, five exposures to the attenuated schistosome vaccine gave greater protection (86%) than three exposures (54%) [22,23].

In general, throughout the vaccination-challenge trial period, multiple exposures of pigs to UVAC developed stronger and more prolonged cellular and humoral immune responses in comparison to a single vaccination. However, there were distinct differences of cytokine and antibody responses between the two immunization protocols. Higher IFN-γ levels were elicited by UVAC immunization or challenge infection. Three vaccinations induced a more significant Th1-type cellular response with higher level of PBMC IFN-γ production after *in vitro *stimulation with schistosome-specific antigens than a single immunization, especially prior to and at the early stage of the challenge infection. This provides evidence that this strong IFN-γ response might contribute to the high level of protection induced by UVAC. In the murine model, IFN-γ is a dominant cytokine that is required to activate pulmonary macrophages which mainly mediate immune elimination of challenge parasites in the lungs [24,25]. Vaccination with irradiated cercariae of *S. mansoni *preferentially induces the accumulation of IFN-γ producing T-cells in the skin and skin-draining lymph nodes (sdLN) of mice, that are then recruited to the lung [26]. Furthermore, a proportion of schistosome-specific Th1 cells generated in the sdLN were likely to become memory cells, and could promote the production of large amount of cytokines and provoke DTH reactions following secondary schistosome challenge [27]. In addition, multiple vaccinations with radiated cercariae also induced the secretion of regulatory IL-10 in accordance with IFN-γ, which has an important role early in the regulation of IL-12-mediated priming of acquired immune responses, and effectively contains excessive inflammation and prevents the development of highly polarized Th1-type responses [28]. IL-4 in the Vac3-Cha and Vac1-Cha groups was generally produced at lower levels. Thus, in pigs vaccinated a single time with attenuated *S. japonicum *cercariae, protection was associated with a IFN-γ dominant cytokine response. In contrast, pigs vaccinated multiple times displayed a strong and mixed IFN-γ/IL-10 response suggesting cell-mediated mechanisms are responsible for the more pronounced protection.

On the other hand, triple UVAC vaccinations elicited stronger IgG responses, including IgG1 and IgG2 isotypes, than a single vaccination. When the ratios of parasite-specific IgG1/IgG2 were analyzed, we found that multiply immunized pigs gradually exhibited higher levels of antigen-specific IgG2 than singly vaccinated animals following challenge infection (Table 1). It is well established that antibodies can play a protective role following multiple vaccinations [29]. Triple vaccinations did not enhance the immunity of B-cell deficient (µMT) mice against *S. mansoni *infection, suggesting that the cell-mediated response was not boosted by multiple exposures to attenuated larvae without the help of antibodies [3]. Additionally, there are reports that IgG antibodies also play a major role in irradiated-vaccine-induced protective immunity in primates. In grivet monkeys vaccinated with a radiation-attenuated *S. mansoni *vaccine, specific IgG levels also peaked after three vaccinations, and there was a clear correlation between the antibody levels at the time of challenge and the protection observed in individual monkeys [22]. In pigs, IFN-γ and IL-12 induced a bias towards IgG2 production while IL-10 up-regulated IgG1; therefore the IgG2 subclass is considered to be associated with a Th1-cell-controlled immune response, whist the IgG1 subclass is associated with a Th2-cell-controlled response [30]. Furthermore, in pigs IgG2 is more effective than IgG1 for the activation of complement [31]. Thus, our trial provides further support that the protective efficacy of multiple UVAC vaccinations against *S. japonicum *infection is partly engendered by theTh1-associated IgG2 subclass of antibodies.

## Conclusion

High levels of protection could be induced in pigs, vaccinated with 400 μw UV-attenuated *S. japonicum *cercariae, against a subsequent challenge infection, and that three vaccinations were possibly more effective than a single vaccination. Moreover, the triple vaccinations evoked a more vigorous IFN-γ response and a stronger antibody-mediated response, especially an increase in the levels of IgG2 antibodies, resulting in higher protective immunity in *S. japonicum *challenged pigs. The radiation-attenuated vaccine, therefore, elicits a multifaceted immune response from which we can derive valuable insights relevant for the future design of novel delivery systems and adjuvants for recombinant and subunit schistosome vaccines.

## Methods

### 1. Animal, parasite and antigen preparation

The study was carried out using 18~20 kg castrated male Landrace/Yorkshire/Duroc crossbred pigs, selected randomly from Topigs Limit Corporation, China. During the entire trial, the pigs were housed together in the farm of the Veterinary Institute of Jiangsu Provincial Agricultural Scientific Academy (Nanjing, China) under controlled sanitary conditions. All pigs were subjected to anthelmintic treatment using albendazole 400 mg twice daily with meals for three days beginning on the 7^th ^day before the experiment. Stool examination showed that all pigs were negative for *S. japonicum*. Sampling procedures, except fecal collection, were performed under intramuscular (i.m.) anaesthetic (3% pentobarbital 1 ml/kg body weight). All experiments involving pigs were performed in accordance with protocols approved by the Institutional Animal Care and Use Committee at Nanjing Medical University.

Cercariae were released from *Oncomelania hupensis *snails, laboratory-infected with a Chinese mainland strain of *S. japonicum*, purchased from the Jiangsu Institute of Parasitic Disease (Wuxi, China). Snails were placed in deionized water and exposed to incandescent light for 3 to 4 hours. Cercariae were collected from the water surface using a 10 μl bacteriological loop and placed on glass cover slips, for percutaneous infection of pigs. *S. japonicum *specific antigens, including soluble adult worm preparations (SWAP) and soluble egg antigen (SEA), were prepared as described elsewhere [32,33] and the protein concentration was determined by BCA protein Assay Kit (Pierce Biotechnology, Inc., IL, USA) [34].

### 2. Experimental schedule

#### 2.1 Preparation of UV-attenuated cercariae (UVAC)

Freshly shed *S. japonicum *cercariae were irradiated by ultraviolet radiation using a portable ultraviolet lamp (Type N16; Konrad Benda, Laborgerate, D-6908 Wiesloch, FRG)at 254 nm with an intensity of 400 μw/cm^2 ^for 1 min.

#### 2.2 Vaccination trials

**Figure 6:**
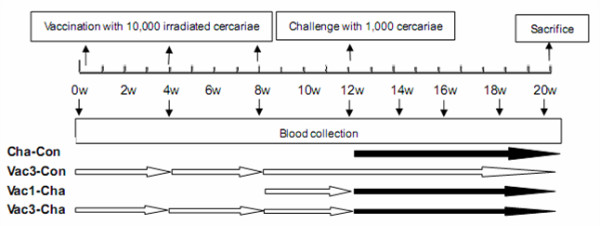
**Experimental schedule for vaccination, challenge and blood collection**. White arrows represent UVAC vaccination. Black arrows represent challenge with normal cercariae.

The experimental procedure is illustrated in Figure 6. The pigs were allocated according to litter origin into four groups of six pigs each. Three groups were vaccinated percutaneously with 10,000 UVAC on their shaved flank skin for 30 min using the cover glass method. The Vac3-Cha and Vac3-Con groups were vaccinated three times at 4-week intervals, and the Vac1-Cha group was vaccinated just one time at 8 weeks. Four weeks after the last vaccination, the Vac3-Cha, Vac1-Cha and Cha-Con groups, were challenged with 1,000 normal cercariae of *S. japonicum*. The groups of Vac3-Cha and Vac1-Cha were set up to evaluate the protective efficacy and investigate the relevant immune events compared to Cha-Con group. Ideally, no mature adult worms and eggs should be observed in the Vac3-Con group if radiation to the cercariae is successful. The inclusion of this Vac3-Con group was to monitor the immunity effect and investigate the corresponding immune response as one reference.

### 3. Parasitological assessments for UVAC-induced protection

#### 3.1 Adult worm recovery and egg counts in liver tissue

All pigs were sacrificed at 8 weeks post-challenge and subjected to portal perfusion. In brief, pigs were intravenously (IV) injected with heparin sulfate (5000 IU) followed by a lethal IV injection of pentobarbital (30 mg/kg). One central longitudinal cut was performed from the sternum to the lower abdomen and selective perfusion of the portal hepatic system and the intestinal mesenteric system was achieved by clamping the vessels supplying blood to other organs and hind legs. The perfusion tube was inserted into the aorta and sodium citrate containing saline was pumped through the portal and mesenteric vessels and the perfusate collected at the punctured portal vein. The perfusate was sieved and worms were collected. After perfusion, the intestinal tract of each pig was examined for residual worms [35,36]. A five gram sample of the left lateral hepatic lobe from each pig was digested in 5% KOH for 18 hours at 37°C and eggs counted in five separate 1 ml subsamples of the KOH solution. The mean count was used to determine eggs per gram (EPG) in the livers of the vaccinated and control pigs [37].

#### 3.2 Fecal egg output

Fresh fecal samples were collected from pigs at one week intervals starting from 4 weeks post-challenge. The filtration and sedimentation Danish Bilharziasis Laboratory (DBL)-technique was used to determine the number of eggs per gram (EPG) in the feces [38]. In brief, a 5 g sample from a homogenized 50~100 g fecal specimen was mixed with saline, shaken and passed through a series of sieves (45, 100, and 400 μm). The fecal material from the 45 μm sieve was washed into a sedimentation glass, filled with saline and left in the dark to sediment. The sediment was centrifuged and resuspended with saline to obtain a volume of 2.25 ml and 150 µl of this solution was mixed with 850 μl 0.9% saline in 1 ml microscope chamber slides. Eggs were counted to obtain the fecal EPG. in the vaccinated and control pigs.

#### 3.3 Vaccine-induced protection

To coordinately compare the protection efficiencies of single or triple vaccinations with UVAC, the vaccine-induced protection was measured using the percentage of worm or egg reduction in vaccinated groups using the formula:

### 4. Immunological parameters

#### 4.1 The levels of schistosome-specific IgG, IgM antibodies and IgG1, IgG2 isotypes

Five ml samples of blood were collected from each pig by precaval venipuncture monthly till challenge and thereafter fortnightly (see Figure 6). The levels of schistosome antigen-specific IgG, IgM, IgG1 and IgG2 antibodies were measured in swine sera by ELISA. In brief, 96-well plates (Costar, Roskilde, Denmark) were coated with SEA (8 µg/ml for IgG and IgM, 15 µg/ml for IgG1 and IgG2) or SWAP (15 µg/ml) at 4°C overnight, then washed twice in PBS-0.05% Tween-20 (PBST) and blocked in PBST-5% skimmed milk at 37°C for 1 hour. Sera samples were diluted 1:100 with PBS and incubated for 2 hours at 37°C. After five additional washes with PBST, IgG and IgM levels were detected using HRP conjugated goat anti-pig IgG (AbD Serotec, Oxford, UK, AHP865P), and goat anti-pig IgM (Serotec, AAI39P) diluted 1:30,000 in PBS and incubated at 37°C for 1 hour. For IgG1 or IgG2 detection, plates were incubated with mouse anti-porcine IgG1 (Serotec, MCA635) or IgG2 (Serotec, MCA636) diluted 1:100 in PBS at 37°C for 1 hour. After washes, plates were incubated at 37°C for 1 hour with 1:10,000 diluted HRP conjugated goat anti-mouse IgG. Finally after five washes with PBST, all the plates were developed with TMB substrate (AMERESCO) for 30 min and the reaction stopped by adding 50 µl 2M H_2_SO_4_. The plates were read at 450 nm, using an ELISA reader (Bio-Rad mod. 550). Each serum sample was tested in triplicate. A serum sample from a pig with *S. japonicum *infection served as a positive control, and a sample from an uninfected pig was used as a negative control.

#### 4.2 Cytokine detection in the culture supernatants of peripheral blood mononuclear cells (PBMC)

20 ml blood samples were collected in heparinized tubes by precaval venipuncture at different time points (Figure 6). Peripheral blood mononuclear cells (PBMC) were isolated using swine lymphocyte separation medium (density: 1.110, Haoyang Bioproduction Limit Corporation, Tianjin, China). PBMC were cultured at 1.5 × 10^6^/ml in RPMI 1640 medium supplemented with penicillin (100 U/ml), streptomycin (100 μg/ml) and 10% fetal calf serum (FBS) in 96-well flat-bottomed microtiter plates (Costar, Roskilde, Denmark) in the presence of 12.5 μg/ml PHA, or 50 μg/ml SEA, or 50 μg/ml SWAP, or complete RPMI 1640 alone in triplicate. PBMC were incubated at 37°C in a humidified atmosphere with 5% CO_2 _for 72 hours and the supernatants were harvested for cytokine determination. IFN-γ, IL-4 and IL-10 were measured using ELISA Kits following the manufacturer's instructions (Invitrogen Corporation, California, USA). The plates were read at 450 nm using an ELISA reader, and the levels of IFN-γ, IL-4 and IL-10 in the supernatants were determined using standard curves constructed using the cytokine standards provided by the manufacturer.

### 5. Statistical analysis

All statistical analysis was performed using SPSS for Windows 13.0. The data were analyzed using one-way analysis of variance (ANOVA). All values presented are means ± SEM. For all tests, *P *values < 0.05 and *P *values < 0.01 were considered significant and highly significant, respectively.

## Competing interests

The authors declare that they have no financial, professional or personal competing interests related to this article. The funding agencies played no role in the design or implementation of the study, analysis or interpretation of the data, or the preparation and submission of the manuscript.

## Authors' contributions

DDL and HWW designed and carried out the animal experiments. DDL and FT participated in testing the immune responses, analyzed the data and drafted the manuscript. YNG, JJW and DHZ participated in testing the immune responses. MJJ and GLW conceived and designed the experiments, analyzed the data, contributed the reagents and materials and helped draft the manuscript. DPM and HPD helped finalize the manuscript. All authors read and approved the final version of the paper.
